# Proteome-wide drug screening using mass spectrometric imaging of bead-arrays

**DOI:** 10.1038/srep26125

**Published:** 2016-05-19

**Authors:** Ying Zhou, Ziying Liu, Kenneth J. Rothschild, Mark J. Lim

**Affiliations:** 1 AmberGen, Inc., 313 Pleasant Street, Watertown, MA 02472, United States; 2 Molecular Biophysics Laboratory, Department of Physics and Photonics Center, Boston University, Boston, MA 02215, United States

## Abstract

A fundamental challenge in the drug discovery process is to develop compounds with high efficacy and minimal side-effects. We describe a new approach to proteome-wide drug screening for detection of on- and off-target binding which combines the advantages of mass spectrometry with microarray technology. The method involves matrix-assisted laser desorption/ionization mass spectrometric imaging (MALDI-MSI) of agarose micro-beads randomly arrayed at high-density in custom micro-well plates. Each bead carries a unique protein target and a corresponding photocleavable mass-tag for coding (PC-Mass-Tag). Compounds bound to specific protein beads and a photo-released coding PC-Mass-Tag are detected simultaneously using MALDI-MSI. As an initial demonstration of this approach, two kinase-targeted drugs, Dasatinib and Brigatinib (AP26113), were simultaneously screened against a model 50-member kinase-bead library. A MALDI-MSI scan performed at the equivalent density of 495,000 beads in the footprint of a microscope slide yielded 100% sensitivity for detecting known strong interactions with no false positives.

An initial step in the drug discovery process depends on identifying and optimizing lead compounds which interact with high affinity and regulate the activity of specific biological drug targets (*e.g.* a protein receptor involved in a disease pathway). However, a second equally important step is to verify that these leads do not interact and interfere with the functioning of any of the vast number of other (non-target) proteins which comprise the human proteome^1^. This second step is critical since there are over 100,000 deaths per year in the U.S. that are caused by serious adverse drug reactions (SADRs), such as through off-target effects^2^,^3^. Furthermore, the selection of lead compounds and their subsequent clinical testing is a long (~10–15 year) and expensive (>$1B) process^4^. On average only 30% of drugs reach Phase I with only 8% of drugs receiving FDA approval^5^. Thus, the ability to perform proteome-wide screening of drug-protein interactions to assist in selecting lead compounds that have minimized off-target effects could result in a much faster and cheaper drug discovery process, as well as safer drugs (complementing but not replacing animal and human studies). Such proteome-wide screening could also be used to repurpose existing drugs^6^,^7^,^8^,^9^.

Microarrays were first developed for genomics more than two decades ago^10^,^11^ and subsequently for proteomics 15 years ago^12^,^13^. Since microarrays facilitate massively parallel analysis of the interaction of prey molecules with thousands to millions of arrayed bait molecules, they are potentially well-suited for proteome-wide drug-protein screening. Protein microarrays can reach densities of >10,000 features on a single chip the size of a microscope slide^14^,^15^ and bead-arrays can reach millions to billions of features in the same footprint^16^,^17^. However, a common feature of these arrays is the use of fluorescence readout and hence the requirement for labeling the prey molecules. In the case where the prey is a drug compound, it is important to avoid labeling which can alter its binding specificity and activity.

Conversely, a variety of effective *label-free* methods exist which can measure drug-protein interactions. Two of the most widely used proximity based assays are fluorescence resonance energy transfer (FRET) (where the unlabeled drug competes for a labeled compound in the protein binding site) and surface plasmon resonance (SPR)^18^. However, these assays are not ideally suited for proteome-wide screening. FRET type assays generally require some *a priori* knowledge of binding or target activation mechanisms and are not easily generalized to the entire proteome (*e.g.* fluorescent tracers are used at the ATP binding site in time-resolved fluorescence resonance energy transfer [TR-FRET] based LanthaScreen® kinase assays^18^). Furthermore, such assays utilize microtiter plates, which although suitable for high throughput screening (HTS), remain impractical for routine proteome-wide screening. For example, although the proteome size (which is larger than human genome) has been difficult to estimate^1^,^15^, it would still require more than a dozen 1,536-well microtiter plates to perform a single screen even against the only 23,500 genes in the human genome^19^. Likewise, existing commercial SPR systems offer limited multiplex capabilities, with Bio-Rad’s ProteOn™ XPR36 instrument offering a 6 × 6 array (36 simultaneous measurements) and the BiacoreTM4000 system offering a 4 × 5 array (reporting throughput of 4,800 measurements but on only 16 targets and requiring 24 hours)^20^. Emerging methods of SPR imaging (SPRi) could increase capacity, with reports of arrays containing approximately 800-features^21^. However, even with expanded SPR multiplex capability, it is difficult for SPR to identify or characterize a binding drug when more than one compound is screened simultaneously.

Mass spectrometry, a central tool in proteomics^22^, has also been used extensively as a *label-free* technology in the drug discovery process^7^. For example, methods using size exclusion or affinity chromatography separation followed by mass spectrometry, including affinity selection mass spectrometry^23^,^24^,^25^,^26^ and frontal affinity chromatography mass spectrometry^26^, enable screening of multiple compounds against single protein targets. Parallel analysis of drug binding to individual proteins each located in a 96 or 384 mini-column format has been reported with these approaches^26^. However, proteome-wide drug screening would require tens of thousands of such parallel columns. Alternatively, a mass spectrometry based chemical proteomics approach uses a competitive binding assay between the free drug of interest and kinases immobilized by broad-selectivity inhibitors^27^. This versatile tool is valuable in drug discovery and as an “in-depth” approach to reveal the mechanisms of action of the inhibitors. However, it involves many experimental steps including protein digestion, identification and quantification. Furthermore, the method is specific to certain protein classes such as kinases since broad-specificity inhibitors are needed to immobilize the targets, and again, cannot be readily generalized to proteome-wide screening.

We report a new, convenient and simple approach for proteome-wide drug screening termed bead-based global proteomic screening (Bead-GPS) which combines the advantages of microarray (bead-array) technology with *label-free* mass spectrometry readout (Fig. 1). Previously, we demonstrated that photocleavable peptides can be utilized as PC-Mass-Tags to encode random bead-arrays. In combination with correlated MALDI-MSI and fluorescence readout, this provided a label-based method for detecting protein-protein molecular interaction such as between a protein library and fluorescently labeled antibodies or other proteins^28^. Here, we demonstrate a *label-free* method for detection of drug-protein interactions that uses PC-Mass-Tags and proteins attached to beads but does not require fluorescence detection. This approach not only allows screening of a drug against a large number of protein targets in a high density microarray format, but has the additional advantage that, unlike SPR, can simultaneously screen multiple drugs against all protein targets owing to the ability to identify each binding drug by mass spectrometry.

## Results and Discussion

### Construction of a model recombinant kinase-agarose-bead library

To demonstrate feasibility of the Bead-GPS approach, a model 50-member recombinant kinase-bead library encoded with PC-Mass-Tags was prepared and validated. To prepare the library, 50 recombinant kinases which had been site-specifically biotinylated at the N-terminus (Carna Biosciences) were attached to 34 μm streptavidin agarose beads. Peptides (7 to 15-mer) labeled at the N-terminus with photocleavable biotin (PC-Biotin)^29^ were used as PC-Mass-Tags. Each kinase-bead species was encoded with a single unique PC-Mass-Tag (see Supplementary Table 1 for all kinases and PC-Mass-Tags). Higher capacity coding for a larger bead library is possible using two or more PC-Mass-Tags on each bead as demonstrated in Supplementary Fig. 1.

Synchronization of matrix-assisted laser desorption/ionization mass spectrometry (MALDI-MS) and fluorescence images as described previously^28^ was used to validate the PC-Mass-Tag coding of individual kinase-beads in the library. Since all 50 kinases contained a common FLAG epitope tag, the bead library was probed with a fluorescently labeled (DyLight650®) anti-FLAG antibody. A specific kinase (LCK) chosen in the library was also *simultaneously* probed with an LCK-specific antibody that was fluorescently labeled with a different fluorophore (Phycoerythrin). After antibody binding, the 50-member kinase-bead library was randomly incorporated into a micro-well plate to form the array (micro-well plates contain approximately 1 million wells in the footprint of a standard microscope slide, each well sized large enough to hold only a single 34 μm bead^28^).

**Figure 2:**
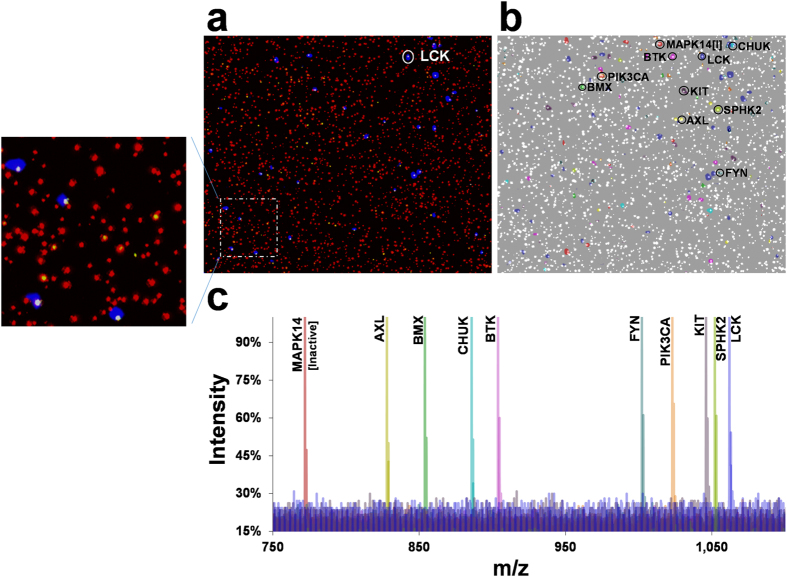
Production of a 50-member kinase bead-library. **(a)** The 50-member kinase bead-library was probed with fluorescent antibodies against both the FLAG epitope (common to all kinases) and a specific kinase (LCK in this example; different fluorescent colors on each antibody). A 2-color fluorescence image overlay is shown of the bead-array where red is the FLAG detection (all kinases) and yellow the specific kinase detection (LCK). The array was also imaged by MALDI-MS where blue is the overlaid MALDI-MS image of the PC-Mass-Tag which codes the LCK beads (aligns with yellow fluorescence). An enlarged view of the area outlined by the dashed square is shown on the left. **(b)** Colorized MALDI-MS images of the same array are shown for 10 kinase bead species, overlaid with the fluorescence image of the FLAG antibody detection (shown as white in this case). **(c)** Color-coded and overlaid MALDI-MS spectra from representative single beads (the beads circled and labeled with kinase gene names in Panels **a**,**b**).

Figure 2a shows a 2-color fluorescence image of the resulting array, whereby red indicates anti-FLAG antibody detection (all kinase-beads) and yellow is the anti-LCK antibody detection (specific kinase-beads). This same array was also imaged by MALDI-MS to detect the PC-Mass-Tag residing on each bead. The blue in Fig. 2a is the overlaid (synchronized) MALDI-MS image of the PC-Mass-Tag species used to code for the LCK kinase beads. As seen, it *correctly* aligns with the yellow fluorescence of the anti-LCK antibody. Note that MALDI-MSI detected approximately 50% of the beads based on visual comparison to the anti-LCK and anti-FLAG fluorescence images, which may be the result of uneven MALDI-MS matrix coating or incomplete matrix penetration into all wells.

Figure 2b shows a colorized MALDI-MS image of the same random bead-array for10 selected PC-Mass-Tags corresponding to 10 different kinase-bead species from the entire 50-member library. The MALDI-MS image was again overlaid onto the common anti-FLAG fluorescence image (with the fluorescence now shown in white). Each discrete MALDI-MS spot aligns with a bead detected by the anti-FLAG antibody. Figure 2c shows color-coded overlaid MALDI-MS spectra from 10 representative *individual* beads selected from the 10 kinase-bead species (see circled beads in Fig. 2a,b). This data confirms that single-bead resolution was obtained and that no bead-to-bead cross-contamination of PC-Mass-Tags occurs, since only a single PC-Mass-Tag species appears in each spectral trace.

### Demonstration of drug-protein interaction screening using Bead-GPS technology

Next we validated the ability of Bead-GPS to detect drug-protein interactions and also to *simultaneously* screen multiple drugs against the entire kinase-bead library. Two model drugs were chosen for this purpose, Dasatinib and Brigatinib. Dasatinib (BMS-354825) is a BCR-ABL and SRC family tyrosine kinase inhibitor used to treat chronic myelogenous leukemia (CML)^30^. Brigatinib (AP26113) is a dual inhibitor of anaplastic lymphoma kinase (ALK) and mutant epidermal growth factor receptor (mEGFR), developed to be used as a second-line drug for non-small cell lung cancer patients that exhibit a drug-resistant mutation in ALK (mALK with L1196M)^31^.

The kinase-bead library was simultaneously treated with both drugs, extensively washed and then incorporated into the micro-well plate to form the random bead-array. MALDI-MSI scanning was then performed on a 5.8 × 6 mm region of the array (in this case, fluorescence imaging is not required). The readout was performed using a SimulTOF 200 Combo MALDI-MS instrument (SimulTOF Systems, Sudbury, MA) which provided high mass resolution (m/Δm = 20,000), a high repetition rate Nd:YLF laser (5 kHz), high digitizer acquisition rate (50–100 pixels/second) and continuous laser raster scanning. The MALDI-MS laser acted to simultaneously desorb/ionize the PC-Mass-Tag and the drugs which non-covalently bind to the proteins attached to each bead. Key parameters for the experiment are listed in Supplementary Table 2. Briefly, the MALDI-MSI scan was performed at 50 μm pixel size over a period of 40 minutes which covered 16,800 micro-wells. By manually enumerating discrete beads (spots) in representative areas of the MALDI-MSI image and comparing to the pixel count for that area, it was determined that on average each bead comprised 1 pixel (which agrees with the 45 μm diameter of the micro-wells^28^ compared to the 50 μm pixel size). Therefore, based on pixel counts, an estimated 9,235 beads were detected by MALDI-MSI in the scanned region (average bead redundancy per kinase of 181). This corresponded to beads detected in 55% of the total micro-wells. At this detected bead density (264/mm^2^) it is estimated that 495,000 total beads would be detected on a single chip the size of a standard 75 × 25 mm microscope slide (note that either the entire array can be used for an experiment or the array can be sectored into sub-arrays using silicone gaskets).

To process the data, the SimulTOF software was first used to detect PC-Mass-Tag and drug peaks in all scanned spectra and calculate the monoisotopic peak area (see Methods). All pixels containing none or more than one detected PC-Mass-Tag were then removed from the dataset, leaving only those pixels with a single PC-Mass-Tag species. For each kinase species (each PC-Mass-Tag), monoisotopic peak area for each drug was averaged for all PC-Mass-Tag-positive pixels for that kinase (average of 181 such pixels, *i.e.* beads, per kinase). To reduce noise, *on a per kinase basis*, pixels where the monoisotopic peak area for the drug fell outside of one standard deviation of the mean were rejected. Furthermore, kinases which had pixel (bead) counts <10% of the average were eliminated due their low number of replicate data points, which resulted in the loss of 3 kinases out of 50 for a 94% success rate.

**Figure 3:**
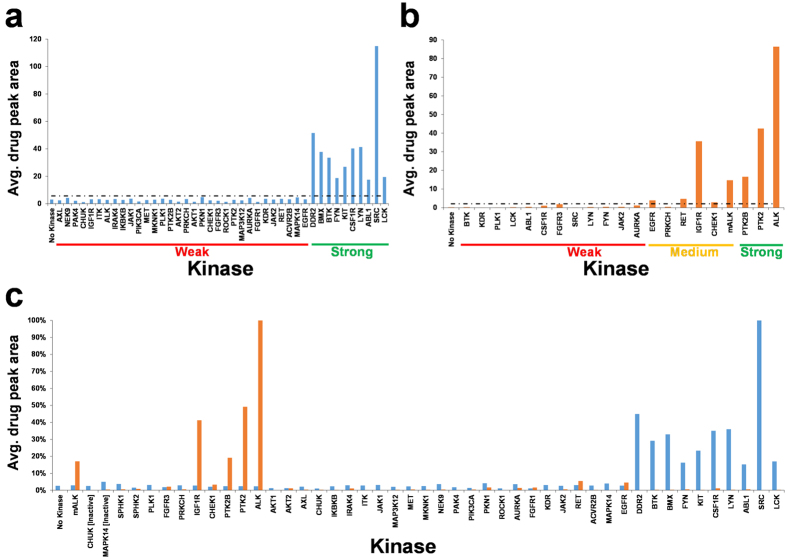
Bead-GPS drug screening with a 50-member kinase bead-array. The 50-member kinase bead-library was treated simultaneously with two drugs, Dasatinib and Brigatinib. The beads were then arrayed and the array scanned by MALDI-MSI. Binding results are expressed as average monoisotopic peak area for the drug on all PC-Mass-Tag positive beads for each kinase. **(a)** Bead-GPS binding results for the drug Dasatinib for each kinase, where the K_d_ values are known. **(b)** Bead-GPS binding results for the drug Brigatinib for each kinase, where the IC_50_ values are known. Drug-kinase interactions with known K_d_ or IC_50_ values are categorized as follows: Weak >100 nM; Medium ≤100 nM but >10 nM; Strong ≤10 nM. The black dotted line is the cutoff for scoring hits, set at 3 standard deviations above the mean of the known weak drug-kinase interactions. **(c)** Bead-GPS binding results for the drugs Dasatinib & Brigatinib for all kinases. Blue Bars are for Dasatinib; Orange Bars are for Brigatinib.

Figure 3 displays the Bead-GPS results for Dasatinib and Brigatinib. For many but not all of the kinases in the library, the K_d_ or IC_50_ values for the drugs are known (see Supplementary Table 1). Therefore, to validate the Bead-GPS results, drug-kinase interactions were categorized following an earlier study^31^ as weak for K_d_ or IC_50_ >100 nM; medium for ≤100 nM and >10 nM; and strong for ≤10 nM. Since Bead-GPS measures binding, K_d_ values were used instead of IC_50_ when available. For Dasatinib, K_d_ values were available from the Drug2Gene database^32^ for 42 kinases. Bead-GPS results for Dasatinib for these kinases are shown in Fig. 3a. The known weak interactions give consistently zero or negligible MALDI-MS Dasatinib signals, while the known strong interactions give a range of strong positive MALDI-MS signals (note there were no known interactions falling into the medium category for Dasatinib). If a cutoff to score hits was set at three standard deviations above the mean for the known weak interactions (black dotted lines in Fig. 3), 100% sensitivity and specificity was achieved for the known strong interactions.

Although the second drug, Brigatinib, is not nearly as well characterized as Dasatinib (and K_d_ values not available), IC_50_ values for 21 of the kinases have been published^31^. Figure 3b shows the Bead-GPS results for these kinases. Using the same cutoff and scoring method as above, 100% sensitivity for the known strong interactions was observed. In the case of medium interactions, an 83% sensitivity and 100% specificity was obtained. The full Bead-GPS binding profile for all kinases and both drugs is shown in Fig. 3c. Importantly, as expected Dasatinib and Brigatinib show *completely* different binding profiles with no overlap. Supplementary Fig. 2 shows representative MALDI-MS spectra from single beads of the drugs along with the PC-Mass-Tags which code for the drug-binding kinases.

Based on the data in Fig. 3, the weakest interaction detected by Bead-GPS of the drug-protein pairs *expected to bind* was ~80 nM (IC_50_). However, future optimizations such as washing steps and studies of more interactions of various strengths will be necessary to more accurately determine the detection limits. It should be emphasized that the magnitude of the Bead-GPS signals among different kinases is not expected to quantitatively correlate with the magnitude of the corresponding K_d_ or IC_50_. For example, if the Bead-GPS signals are plotted against K_d_ or IC_50_ for all kinases, a linear correlation is not found. Factors which would tend to produce MALDI-MS intensities which do not directly correlate with binding strength include different amounts of target protein on the different bead species and differential extraction/elution of drugs from the various bead species (by matrix compound and laser energy). We propose that this approach would be most useful as a method to initially screen for potential off-target interactions (hits). Once these hits are determined, they would be further validated and analyzed with existing quantitative and kinetic methods such as FRET and SPR assays which are better suited for more in-depth analysis of smaller numbers of protein targets.

## Conclusion

In summary, we demonstrate feasibility of a simple, label-free, mass spectrometric array-based approach (Bead-GPS) for rapidly screening drug-protein interactions that can be scaled for proteome-wide studies. Bead-GPS has the added advantage that multiple drugs may be *simultaneously* screened against the entire protein library, for full drug-by-library multiplexing. In the future, an entire biotinylated kinome- or proteome-scale library could be produced using standard recombinant protein practices^33^, analogous to commonly used polyhistidine- and GST-tagged proteins which have been produced on a proteome-scale^13^. We estimate that for a micro-well plate the size of a microscope slide such as used here, roughly 10,000 unique proteins, or 20 sub-arrays each containing for example the entire kinome (estimated at about 500 kinases^34^), could be analyzed with a 50-fold bead redundancy. High capacity coding of the array can be achieved by combinatorial PC-Mass-Tag schemes^28^. For example, for a library encoded with only two PC-Mass-Tags per bead species, only 54 unique PC-Mass-Tags are needed to provide 1,431 unique codes (see Supplementary Fig. S1). This is more than sufficient for the entire human kinome. Similarly, a library encoded with three PC-Mass-Tags per bead species using only 54 unique PC-Mass Tags is sufficient to encode 24,804 different proteins. Alternatively, a library encoded with only two PC-Mass-Tags per bead using 150 unique PC-Mass-Tags species, is sufficient to encode 11,175 proteins.

## Methods

### Preparation of PC-Mass-Tags

Peptides of unique mass were chemically modified on their N-terminus with PC-Biotin using an N-hydroxysuccinimide (NHS)-activated PC-Biotin labeling reagent^29^ (AmberGen Inc., Watertown, MA). The peptide was prepared at 5 mg/mL in 100 mM sodium bicarbonate and reacted overnight (with mixing) with equimolar amounts of the reagent. The resultant PC-Mass-Tags were used without further purification. Because the NHS-activated labeling reagents react only with primary amines, selective labeling of the N-terminus is achieved^35^.

### Preparation of PC-Mass-Tag streptavidin agarose beads

Preparation of non-fluorescent and fluorescent “Sync Beads” and loading PC-Mass-Tags to the beads has been previously described^35^. Briefly, the fluorescent “Sync Beads” were prepared by attaching fluorescent streptavidin to NHS-activated 34 μm agarose beads (NHS HP SpinTrap, GE Healthcare Life Sciences). Fluorescent streptavidin was produced from the reaction of Streptavidin (Thermo Fisher Scientific) and 2 molar equivalents of Alexa Fluor® 647 carboxylic acid, succinimidyl ester (Invitrogen, Carlsbad, CA, USA). The product was purified using Illustra™ NAP™-5 G-25 Sephadex™ columns (GE Healthcare Life Sciences) according to the manufacturer’s instructions. For non-fluorescent streptavidin agarose beads, commercially available 34 μm beads were used (Streptavidin Sepharose High Performance, GE Healthcare Life Sciences, Piscataway, NJ, USA). PC-Mass-Tags listed in Supplementary Table 1 were used. The PC-Mass-Tags were mixed with the streptavidin beads for 30 min in tris buffered saline with 0.05% (v/v) Tween-20 (TBS-T), followed by washing steps using TBS-T. Beads were stored at 4 °C in tris buffered saline (TBS) or used immediately for subsequent steps. In the case of double PC-Mass-Tags, two PC-Mass-Tags well resolved (mass difference >2.5 Da) in the mass spectrum were incubated with the beads simultaneously at the same concentration as the amount that was used to generate the single-tag beads.

### Construction of PC-Mass-Tag encoded protein-beads

Site specific biotinylated (BTN) protein kinases (Carna Biosciences, Framingham, MA, USA) were loaded to the PC-Mass-Tag beads through biotin-streptavidin interaction. The enzymatic reaction generated BTN-kinase which has a single biotinylation at the N-terminus region to avoid interference with compound binding and to maintain the kinase activity and an overall functional structure. Each PC-Mass-Tag with a unique *m/z* in the mass spectrum serves as the code indicating each individual kinase bead species. In this study, 50 bead species were constructed. Typically, 10 μg of BTN-kinase was incubated with 1 μL of PC-Mass-Tag streptavidin beads in TBS-T, 1% (w/v) BSA for 30 min and washed with TBS-T.

### Incubation of compound library with protein-bead library

A pool of 50 kinase-beads and one control PC-Mass-Tag bead species without any kinases was generated by combining ~5000 beads/species together in TBS-T, 1% (w/v) BSA. The beads were washed and re-suspended in 800 μL TBS-T, 1% (w/v) BSA. The compounds—Dasatinib (LC Labs, Woburn, MA, USA) and Brigatinib (AP26113) (Selleckchem, Houston, TX, USA) were dissolved in DMSO (10 mM) and further diluted with the incubation solution before use. The drugs were mixed and incubated with the protein-bead library at a final concentration of 0.25 μM for each compound for 1.5 hours at room temperature, followed by TBST-T wash.

### Forming random bead-arrays

Custom fiber optic bead-array micro-well plates were manufactured for AmberGen, Inc. by INCOM USA, Inc. (Charlton, MA, USA). The 1 × 75 × 25 mm plates contained ~1 million hexagonally packed fiber optic wells created from 50 μm fibers etched to 40 μm depth. The fiber cladding yields wells of approximately 45 μm i.d. For optimal performance in MALDI-MSI, plates were coated by sputtering on indium tin oxide (ITO) with sheet resistance (Rs) of 50 ohms/sq or less (ThinFilms, Inc., Hillsborough, NJ, USA). The plates were pre-hydrated and assembled into an AHC1×16 Microarray Hybridization Cassette (ArrayIt® Corporation, Sunnyvale, CA, USA) which subdivided the plate into 16 square sub-array zones, each zone measuring 7.5 mm × 7.5 mm.

Prior to loading the beads onto the plate, a wash step with mass spectrometry grade water was added to remove the salts from the beads. Typically, 100 μL of the bead suspension (~2.8 × 10^4^ beads) were loaded to each chamber of the Microarray Hybridization Cassette. Centrifugation at 1430 *g* was performed when the beads were settled into the micro-wells. The fluid supernatant was discarded. The plate was then washed using water. The plate was allowed to dry overnight at room temperature and covered from light. When fluorescent dye was used, a fluorescence image of the bead-arrays was acquired using a GenePix 4200 microarray scanner (Molecular Devices LLC, Sunnyvale, CA, USA).

Photocleavage of the PC-Mass-Tags from the beads was performed by irradiating the plate from above for 5 min with 365 nm UV light using a Blak-Ray lamp (model XX-15; UVP, Upland, CA, USA), at a 5 cm distance (the power output under these conditions was approximately 2.6 mW/cm^2^ at 360 nm, 1.0 mW/cm^2^ at 310 nm and 0.16 mW/cm^2^ at 250 nm).

### MALDI-MSI and data analysis

A mixture of 2,5-Dihydroxybenzoic acid (DHB) (10 mg/mL) and α-cyano-4-hydroxycinnamic acid (CHCA) (5 mg/mL) (Sigma-Aldrich, St. Louis, MO, USA) in 70% methanol (0.1% trifluoro acetic acid and 0.1% phosphoric acid) was sprayed to provide a thin and uniform film of matrix using the HTX TM-Sprayer™ (HTXImaging by HTX Technologies, LLC, Carrboro, NC, USA). The spray parameters were: nozzle XY velocity of 800 mm/min, 18 passes, 0.1 mL/min matrix flow rate, nozzle temperature at 30 °C and track spacing of 3 mm.

MALDI-MSI was performed using an AB Sciex 4800 *Plus* MALDI-TOF/TOF mass spectrometer with a Nd/YAG pulsed laser (355 nm) (AB Sciex, Foster City, CA) and a SimulTOF 200 Combo MALDI TOF mass spectrometer (SimulTOF Systems, Sudbury, MA) in the positive ion operating in reflector mode. For AB Sciex 4800 mass spectrometry, images were acquired with a scan raster of 40 μm steps and 100 laser shots per pixel. For SimulTOF 200 Combo mass spectrometry, all imaged areas were collected using a laser repetition rate of 1 kHz, 10 μJ laser power, 100 laser shots/spectrum, and step size of 25 μm between raster lines.

Acquisition of the MALDI-MSI using AB Sciex 4800 *Plus* was achieved using the public domain software 4000 Series Imaging (Novartis & Applied Biosystems, Markus Stoeckli), which works with the native software on the mass spectrometer, and the images analyzed using the software TissueView^TM^ (AB Sciex). SimulTOF 200 MALDI-MSI was acquired using SimulTOF software: Wizard, Controller and Plate Editor. The data analysis was performed using the SimulTOF Viewer. For peak detection, the signal-to-noise threshold was set to three and the mass tolerance was set to ±0.5 Da.

Although the 100 (AB Sciex 4800) or 50 (SimulTOF) μm diameter laser beam of the MALDI-MS instrument is larger than the actual diameter of wells and individual beads, it is still possible to resolve individual beads by performing imaging in incremental steps that are smaller than the beam diameter. This oversampling approach has been proved to be ideal for MALDI-MSI experiments due to the higher spatial resolution it provides^36^.

Fluorescence imaging of the plates, as detailed earlier^35^, could also be performed after MALDI-MSI. This is particularly useful as the matrix provides some auto-fluorescence when excited with the 488-nm laser (fluorescein channel), thereby allowing visualization of the region scanned by MALDI-MSI, observed as the zone of matrix depletion.

## Additional Information

**How to cite this article**: Zhou, Y. *et al*. Proteome-wide drug screening using mass spectrometric imaging of bead-arrays. *Sci. Rep.* **6**, 26125; doi: 10.1038/srep26125 (2016).

## Supplementary Material

Supplementary Information

## Figures and Tables

**Figure 1:**
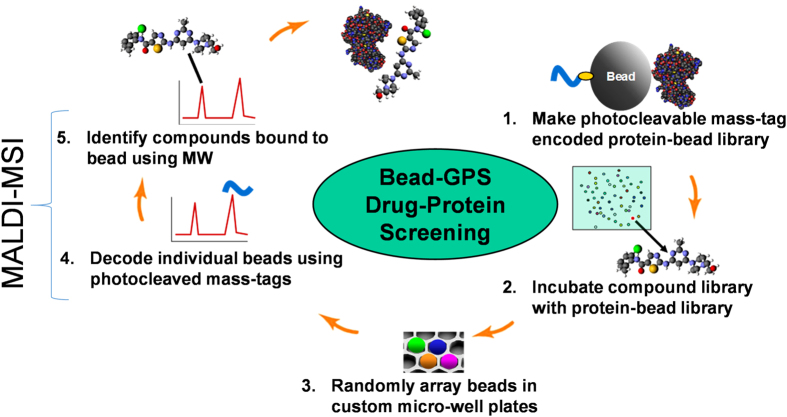
Major steps in proteome-wide drug-protein screening using Bead-GPS. Unlike conventional HTS methods, Bead-GPS enables rapid, proteome-wide screening of entire compound libraries against large protein libraries enabling both on- and off-target drug interactions to be identified. In the example shown, the kinase-directed drug Dasatinib (structure shown in at top of figure) interacts with a particular protein residing on a bead in the array. MALDI-MSI scanning of the bead-array is used to both simultaneously decode the beads and detect the bound drugs. Blue curved line represents a PC-Mass-Tag which is released by the MALDI-MS laser; Yellow oval represents a photocleavable linker which in this case was PC-Biotin.

## References

[b1] Kim M. S. . A draft map of the human proteome. Nature 509, 575–581, doi: 10.1038/nature13302 (2014). 24870542 PMC4403737

[b2] Giacomini K. M. . When good drugs go bad. Nature 446, 975–977, doi: 10.1038/446975a (2007). 17460642

[b3] Yamanishi Y., Pauwels E. & Kotera M. Drug side-effect prediction based on the integration of chemical and biological spaces. J Chem Inf Model 52, 3284–3292, doi: 10.1021/ci2005548 (2012). 23157436

[b4] Hughes J. P., Rees S., Kalindjian S. B. & Philpott K. L. Principles of early drug discovery. British journal of pharmacology 162, 1239–1249, doi: 10.1111/j.1476-5381.2010.01127.x (2011). 21091654 PMC3058157

[b5] DiMasi J. A., Hansen R. W. & Grabowski H. G. The price of innovation: new estimates of drug development costs. J Health Econ 22, 151–185, doi: 10.1016/S0167-6296(02)00126-1 (2003). 12606142

[b6] Adams J. C. . A mapping of drug space from the viewpoint of small molecule metabolism. PLoS Comput Biol 5, e1000474, doi: 10.1371/journal.pcbi.1000474 (2009). 19701464 PMC2727484

[b7] Bakhtiar R. & Nelson R. W. Mass spectrometry of the proteome. Mol Pharmacol 60, 405–415 (2001). 11502869

[b8] Bantscheff M., Scholten A. & Heck A. J. Revealing promiscuous drug-target interactions by chemical proteomics. Drug Discov Today 14, 1021–1029, doi: 10.1016/j.drudis.2009.07.001 (2009). 19596079

[b9] Oprea T. I. & Mestres J. Drug repurposing: far beyond new targets for old drugs. AAPS J 14, 759–763, doi: 10.1208/s12248-012-9390-1 (2012). 22826034 PMC3475856

[b10] Chee M. . Accessing genetic information with high-density DNA arrays. Science 274, 610–614 (1996). 8849452 10.1126/science.274.5287.610

[b11] Fodor S. P. . Light-directed, spatially addressable parallel chemical synthesis. Science 251, 767–773 (1991). 1990438 10.1126/science.1990438

[b12] MacBeath G. & Schreiber S. L. Printing proteins as microarrays for high-throughput function determination. Science 289, 1760–1763. (2000). 10976071 10.1126/science.289.5485.1760

[b13] Zhu H. . Global analysis of protein activities using proteome chips. Science 293, 2101–2105, doi: 10.1126/science.1062191 (2001). 11474067

[b14] International Human Genome Sequencing Consortium: Finishing the euchromatic sequence of the human genome. Nature 431, 931–945, doi: 10.1038/nature03001 (2004). 15496913

[b15] Harrison P. M., Kumar A., Lang N., Snyder M. & Gerstein M. A question of size: the eukaryotic proteome and the problems in defining it. Nucleic Acids Res 30, 1083–1090 (2002). 11861898 10.1093/nar/30.5.1083 PMC101239

[b16] Leamon J. H. . A massively parallel PicoTiterPlate based platform for discrete picoliter-scale polymerase chain reactions. Electrophoresis 24, 3769–3777, doi: 10.1002/elps.200305646 (2003). 14613204

[b17] Michael K. L., Taylor L. C., Schultz S. L. & Walt D. R. Randomly ordered addressable high-density optical sensor arrays. Anal. Chem. 70, 1242–1248 (1998). 9553489 10.1021/ac971343r

[b18] Mason J. L. . Comparison of LanthaScreen Eu kinase binding assay and surface plasmon resonance method in elucidating the binding kinetics of focal adhesion kinase inhibitors. Assay Drug Dev Technol 10, 468–475, doi: 10.1089/adt.2012.453 (2012). 22690705

[b19] Marian A. J. Sequencing your genome: what does it mean? Methodist Debakey Cardiovasc J 10, 3–6 (2014). 24932355 10.14797/mdcj-10-1-3 PMC4051326

[b20] Helmerhorst E., Chandler D. J., Nussio M. & Mamotte C. D. Real-time and Label-free Bio-sensing of Molecular Interactions by Surface Plasmon Resonance: A Laboratory Medicine Perspective. Clin Biochem Rev 33, 161–173 (2012). 23267248 PMC3529553

[b21] Lausted C., Hu Z. & Hood L. Quantitative serum proteomics from surface plasmon resonance imaging. Mol Cell Proteomics 7, 2464–2474, doi: 10.1074/mcp.M800121-MCP200 (2008). 18678562

[b22] Nesvizhskii A. I. Protein identification by tandem mass spectrometry and sequence database searching. Methods Mol. Biol. 367, 87–119, doi: 10.1385/1-59745-275-0:87 (2007). 17185772

[b23] Annis D. A., Nickbarg E., Yang X., Ziebell M. R. & Whitehurst C. E. Affinity selection-mass spectrometry screening techniques for small molecule drug discovery. Curr Opin Chem Biol 11, 518–526, doi: 10.1016/j.cbpa.2007.07.011 (2007). 17931956

[b24] Jonker N., Kool J., Irth H. & Niessen W. M. Recent developments in protein-ligand affinity mass spectrometry. Anal Bioanal Chem 399, 2669–2681, doi: 10.1007/s00216-010-4350-z (2011). 21058031 PMC3043251

[b25] Muckenschnabel I., Falchetto R., Mayr L. M. & Filipuzzi I. SpeedScreen: label-free liquid chromatography-mass spectrometry-based high-throughput screening for the discovery of orphan protein ligands. Anal Biochem 324, 241–249 (2004). 14690688 10.1016/j.ab.2003.09.040

[b26] Ng E. S. . High-throughput screening for enzyme inhibitors using frontal affinity chromatography with liquid chromatography and mass spectrometry. Analytical chemistry 77, 6125–6133, doi: 10.1021/ac051131r (2005). 16194069

[b27] Bantscheff M. . Quantitative chemical proteomics reveals mechanisms of action of clinical ABL kinase inhibitors. Nat Biotechnol 25, 1035–1044, doi: 10.1038/nbt1328 (2007). 17721511

[b28] Lim M. J., Liu Z., Braunschweiger K. I., Awad A. & Rothschild K. J. Correlated MALDI-MS and Fluorescent Imaging of Photocleavable Peptide-Coded Random Bead-Arrays. Rapid Communications in Mass Spectrometery 28, 49–62 (2014). 10.1002/rcm.6754 PMC3894740 24285390

[b29] Olejnik J., Sonar S., Krzymanska-Olejnik E. & Rothschild K. J. Photocleavable Biotin derivatives: A Versatile Approach for the Isolation of Biomolecules. Proceedings of the National Academy of Science (USA) 92, 7590–7594 (1995). 10.1073/pnas.92.16.7590 PMC41385 7638235

[b30] Lombardo L. J. . Discovery of N-(2-chloro-6-methyl- phenyl)-2-(6-(4-(2-hydroxyethyl)- piperazin-1-yl)-2-methylpyrimidin-4- ylamino)thiazole-5-carboxamide (BMS-354825), a dual Src/Abl kinase inhibitor with potent antitumor activity in preclinical assays. J Med Chem 47, 6658–6661, doi: 10.1021/jm049486a (2004). 15615512

[b31] Katayama R. . Therapeutic strategies to overcome crizotinib resistance in non-small cell lung cancers harboring the fusion oncogene EML4-ALK. Proc. Natl. Acad. Sci. USA 108, 7535–7540, doi: 10.1073/pnas.1019559108 (2011). 21502504 PMC3088626

[b32] Roider H. G. . Drug2Gene: an exhaustive resource to explore effectively the drug-target relation network. BMC Bioinformatics 15, 68, doi: 10.1186/1471-2105-15-68 (2014). 24618344 PMC4234465

[b33] Kitagawa D., Gouda M. & Kirii Y. Quick evaluation of kinase inhibitors by surface plasmon resonance using single-site specifically biotinylated kinases. J Biomol Screen 19, 453–461, doi: 10.1177/1087057113506051 (2014). 24080257

[b34] Yan S. F., King F. J., Zhou Y., Warmuth M. & Xia G. Profiling the kinome for drug discovery. Drug Discov Today Technol 3, 269–276, doi: 10.1016/j.ddtec.2006.09.012 (2006). 24980528

[b35] Lim M. J., Liu Z., Braunschweiger K. I., Awad A. & Rothschild K. J. Correlated matrix-assisted laser desorption/ionization mass spectrometry and fluorescent imaging of photocleavable peptide-coded random bead-arrays. Rapid Commun Mass Spectrom 28, 49–62, doi: 10.1002/rcm.6754 (2014). 24285390 PMC3894740

[b36] Spraggins J. M. & Caprioli R. M. High-speed MALDI-TOF imaging mass spectrometry: rapid ion image acquisition and considerations for next generation instrumentation. J Am Soc Mass Spectrom 22, 1022–1031, doi: 10.1007/s13361-011-0121-0 (2011). 21953043 PMC3514015

