# Supplementary material for: Proteome-wide drug screening using mass spectrometric imaging of bead-arrays
Source: Sci Rep. 2016 May 19;6:26125. doi: 10.1038/srep26125 (PMC4872124; doi:10.1038/srep26125)
Supplement: Supplementary Information [file srep26125-s1.pdf]

Proteome-wide drug screening using mass spectrometric imaging of bead-arrays

Supplementary information

Ying Zhou[1], Ziying Liu[1], Kenneth J. Rothschild[1,2*] and Mark J. Lim[1*]

[1]AmberGen, Inc.
313 Pleasant Street
Watertown, MA 02472
and
[2]Molecular Biophysics Laboratory
Department of Physics and Photonics Center
Boston University
Boston, MA 02215

*Address all correspondence to either Mark J. Lim or Kenneth J. Rothschild, AmberGen, Inc., 313 Pleasant Street, Watertown, MA 02472, mlim@ambergen.com or krothschild@ambergen.com.

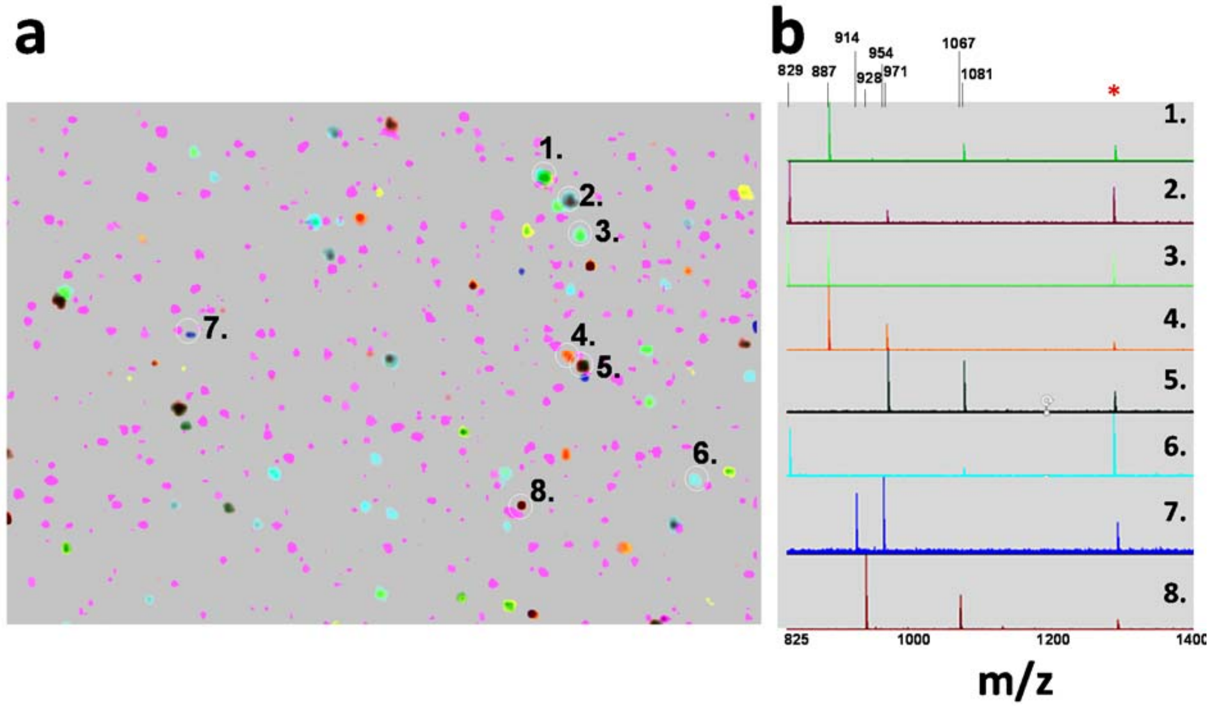

Supplementary Figure 1. **High capacity combinatorial PC-Mass-Tag barcoding of bead libraries. (a)** Color-coded MALDI-MS image of a bead-array containing 85 unique bead species coded with 2 PC-Mass-Tags each (54 unique PC-Mass-Tags used in total). For simplicity, the image was created by assigning unique colors to only 8 of the 54 PC-Mass-Tags (the overlay of 2 colors per bead species also creates a unique color/shade), with the remainder of the PC-Mass-Tags all colored pink. However, all 85 unique bead species were detected in the array region shown. **(b)** Color-coded spectra are shown for 8 representative single beads (the beads noted with white circles and numbered in Panel a). Masses of the 8 different tags are indicated at the top of the compiled spectra, rounded to the nearest integer for simplicity. The red asterisk indicates a control peptide spiked into the matrix solution which is sprayed over the entire bead-array. The numbering of each spectrum corresponds to the bead numbering in Panel A. As an example metric to demonstrate correct pairing of PC-Mass-Tags on the beads in the array, a pixel-by-pixel linear regression correlation between the grayscale MALDI-MS images of 2 PC-Mass-Tags expected to be found uniquely on only 1 bead species (i.e. no other bead species in the library contains either of these 2 mass-tags) yielded an R = 0.86. Conversely, regression analysis of 2 unique PC-Mass-Tags which were used for coding beads in the array, but were not paired on any of the 85 different bead species, returned an R = 0.02. Note that even at this coding level, >1,000 unique codes can be created, more than sufficient to code an entire kinome comprised of the approximate 500 kinases in the human proteome.

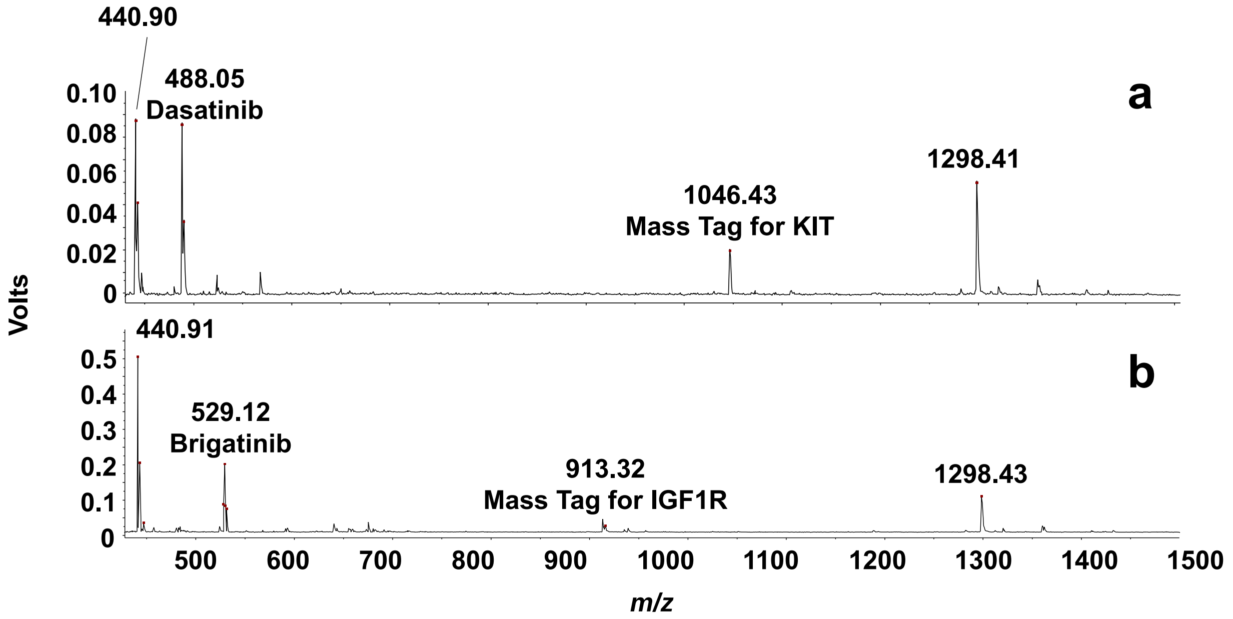

Supplementary Figure 2. Representative MALDI-MS spectra from single beads of the drugs along with the PC-Mass-Tags which code for the drug-binding kinases. (a) Dasatinib and PC-Mass-Tag for KIT shown in one mass spectrum. **(b)** Brigatinib and PC-Mass-Tag for IGF1R shown in one mass spectrum. Note that m/z at 440.9 was a matrix signal and m/z at 1298.4 was the mass signal for an internal standard added in the matrix. These two peaks were used for mass calibration.

Supplementary Table 1 Kinase library, drug K_d, IC_{50} and PC-Mass-Tag coding.

Kinase	K_d (Dasatinib)	IC_{50} (Brigatinib)	PC-Mass-Tag Peptide	PC-Mass-Tag MW (Monoisotopic)
mALK [L1196M Mutant]	No Data	$^{\Delta}$1.61E-08	TRNYEVRAVL	1,219.7
CHUK [Inactive]	No Data	No Data	EAISPPDAASAAPLR	1,464.8
MAPK14 [Inactive]	No Data	No Data	LRRASLG	771.5
SPHK1	No Data	No Data	RGREPLEVFISA	1,372.8
SPHK2	No Data	No Data	FLRGRAYGL	1,051.6
PLK1	1.00E-05	6.11E-07	APRGRFYSL	1,065.6
FGFR3	1.00E-05	3.58E-07	EAISPPDSASAAPLR	1,480.8
PRKCH	1.00E-05	9.50E-08	RTFSDLWKLL	1,277.7
IGF1R	1.00E-05	*3.36E-08	RGYGYQGL	912.5
CHEK1	1.00E-05	3.00E-08	EAISPPDGASAAPLR	1,450.7
PTK2B	1.00E-05	*1.35E-08	APRYRFYSL	1,171.6
PTK2	1.00E-05	*9.65E-09	EAISPPDDASAAPLR	1,508.8
ALK	1.00E-05	*1.81E-09	RGYPYQGL	952.5
AKT1	1.00E-05	No Data	DRVYYHPFHL	1,345.7
AKT2	1.00E-05	No Data	TRNYPVRAVL	1,187.7
AXL	1.00E-05	No Data	PPGASPFR	827.4
CHUK	1.00E-05	No Data	PPGESPFR	885.4
IKBKB	1.00E-05	No Data	RGYHYQGL	992.5
IRAK4	1.00E-05	No Data	RGYEYQGL	984.5
ITK	1.00E-05	No Data	RGYAYQGL	926.5
JAK1	1.00E-05	No Data	RGYRYQGL	1,011.5
MAP3K12	1.00E-05	No Data	EAISPPDHASAAPLR	1,530.8
MET	1.00E-05	No Data	APRLRNYSL	1,088.6
MKNK1	1.00E-05	No Data	APRLRRYSL	1,130.7
NEK9	1.00E-05	No Data	RYPFPGP	832.4
PAK4	1.00E-05	No Data	RPPGFSPL	869.5
PIK3CA	1.00E-05	No Data	WQPPRARI	1,022.6
PKN1	1.00E-05	No Data	RGRETFSDLWK	1,393.7
ROCK1	1.00E-05	No Data	RGRTFSDLWKLL	1,490.8
AURKA	9.30E-06	1.46E-07	TRNYRVRAVL	1,246.7
FGFR1	3.70E-06	No Data	EAISPPDRASAAPLR	1,549.8
KDR	2.90E-06	8.15E-07	APRARFYSL	1,079.6
JAK2	1.00E-06	1.54E-07	RGYNYQGL	969.5
RET	7.30E-07	6.50E-08	APRERFYSL	1,137.6
ACVR2B	5.70E-07	No Data	TRNYGVRAVL	1,147.7
MAPK14	4.95E-07	No Data	RYPFPGPP	929.5
EGFR	1.20E-07	*8.10E-08	EAISPPDEASAAPLR	1,522.8
DDR2	3.20E-09	No Data	EAISPPDYASAAPLR	1,556.8
BTK	1.40E-09	*9.72E-07	PPGFSPFR	903.5
BMX	1.40E-09	No Data	PPGPSPFR	853.4
FYN	7.90E-10	1.98E-07	RSFLLRNP	1,001.6
KIT	6.20E-10	No Data	APRLRAYSL	1,045.6
CSF1R	5.80E-10	3.58E-07	SFLLRNP	845.5
LYN	5.70E-10	2.41E-07	APRLRPYSL	1,071.6
ABL1	5.30E-10	5.00E-07	DRVYGHPFHL	1,239.6
SRC	2.10E-10	3.29E-07	IPSINVHHY	1,078.6
LCK	2.00E-10	5.12E-07	APRLRSYSL	1,061.6
FLT3	1.00E-05	*4.37E-09	EGVNDNPEGFFSAR	1,537.7
MAP2K1 [Inactive]	No Data	No Data	APRLRHYSL	1,111.6
MAP3K5	1.00E-05	No Data	APRLREYSL	1,103.6

Sources for IC_{50} were from Katayama, R. *et al.* as well as measurements by Carna Biosciences. *Some IC_{50} data values were the average of these two sources as indicated by *. The symbol $^{\Delta}$ represents IC_{50} values which were provided by Carna Biosciences only. The remaining IC_{50} values without any labels were from measurements reported by Katayama, R. *et al.* only. K_d were from the Drug2Gene database. See main manuscript for full references.

Supplementary Table 2

Key parameters and results of the 50-kinase and 2-drug Bead-GPS screening experiment

Parameter	Result
Dimensions of scanned region (area)	5.8 x 6 mm (35 mm^2)
Wells in scanned region (wells/mm^2)	16,800 (480)
Beads detected in scanned region by MALDI-MSI (beads detected/mm^2)	9,235 (264)
Beads detected as % of total wells	55%
Average bead redundancy per each kinase species	181
MALDI-MSI pixel size (average pixels/bead)	50 µm (1)
Scan time for region	40 min
Extrapolated total beads detected on an entire chip (25 x 75 mm = 1,875 mm^2)	495,000
Bead diameter	34 µm
Well dimensions	45 µm i.d. and 40 µm depth
